# Twenty-Five Years of FDA Prescription-to-Nonprescription Switch Approvals (2001–2025)

**DOI:** 10.1007/s43441-026-00972-6

**Published:** 2026-04-25

**Authors:** Liudmila Iamukova

**Affiliations:** School of Pharmacy, University of California San Diego, La Jolla, USA

**Keywords:** Prescription-to-nonprescription switch, FDA, Over-the-counter drugs, Self-care, ACNU, Drug accessibility, Public health

## Abstract

The U.S. Food and Drug Administration Prescription-to-Nonprescription (Rx-to-OTC) switch pathway enables consumers to access safe and effective medications without healthcare supervision. Data were extracted from the FDA Prescription-to-Nonprescription Switch List and corresponding New Drug Applications available through Drugs@FDA. All switches approved between 2001 and 2025 and listed in the FDA registry at the time of extraction were analyzed by active ingredient, therapeutic area, indication, year, route of administration, dosage form, strength, and switch type. A total of 45 Rx-to-OTC switches representing 30 unique active ingredients were identified. The Allergy/Respiratory category accounted for 56% of all approvals, followed by Ophthalmology (11%) and Gastrointestinal (11%). Oral formulations were most frequent (58%), with nasal (16%), ophthalmic (11%), topical (11%), and transdermal (4%) routes representing smaller shares. Full switches comprised 64% of all cases, partial switches represented 27%, and direct nonprescription NDAs listed within the registry 9%. From 2001 to 2025, the FDA’s Rx-to-OTC switches demonstrate sustained activity in well-established therapeutic areas with gradual expansion into categories of broader public health relevance. Implementation of the 2024 Additional Conditions for Nonprescription Use framework may influence future switch considerations by permitting structured safeguards beyond traditional labeling to support safe consumer self-selection.

## Introduction

A sponsor of a prescription drug may request a change in its marketing status to make the product available without a prescription. This regulatory process, known as Rx-to-OTC switch, is conducted under the New Drug Application (NDA) framework of the U.S. Food and Drug Administration (FDA).

The sponsor initiates the change by submitting an efficacy supplement to an approved NDA or, in some cases, a new 505(b)(2) application. Depending on the scope of the submission, an Rx-to-OTC switch may be full, in which all conditions of use for the product transition to nonprescription status, or partial, in which only certain indications, doses, or populations are approved for over-the-counter availability while others remain prescription-only. The application must include clinical efficacy and safety data, post-marketing surveillance findings, and consumer-use evidence, such as label comprehension, self-selection, and actual-use studies, demonstrating that patients can safely and effectively use the drug without healthcare supervision. The FDA grants approval when it determines that prescription-only status is no longer required to protect public health, and that the product is safe and effective for self-medication according to the proposed labeling [1]. Against this framework, Rx-to-OTC switches have expanded access to therapies historically used for physician-managed conditions, while preserving safeguards through evidence-based labeling and, where appropriate, product-specific limitations.

The Rx-to-OTC pathway has been examined in prior academic literature from clinical, regulatory, and public health perspectives. Early analyses emphasized the balance between expanding consumer access and maintaining safeguards for appropriate use [2]. Subsequent scholarship has highlighted both the evidentiary complexity and policy considerations surrounding certain high-profile switches, including emergency contraception and other socially sensitive categories [3, 4]. More recent evaluations have characterized long-term switch trends and regulatory patterns in the United States [5].

The present study examines FDA-listed switches from 2001 to 2025, characterizing switch type, alongside active ingredients, indications, routes, dosage forms, doses, and approval dates.

## Materials and methods

Data for this analysis were obtained from the FDA Prescription-to-Nonprescription Switch List [6], which provides information on NDAs that have undergone conversion from prescription to nonprescription status in the United States. The FDA switch list is publicly available and continuously updated to reflect approved Rx-to-OTC transitions. Each entry in the list links to the original NDA information available through the FDA’s Drugs@FDA database [7], which contains detailed regulatory documentation for every marketed product. The switch list was accessed in July 2025 and reviewed. The analysis was limited to NDAs and supplemental NDAs explicitly listed in the list at the time of data extraction. Historical switch events not reflected within the current FDA registry were not independently reconstructed from external sources in order to preserve dataset consistency and reproducibility. Data were manually curated and for each Rx-to-OTC switch, the following parameters were extracted directly from the respective NDA files and FDA public summaries: active ingredients, drug name, indication for switch, switch year, route of administration, dosage form, strength, switch type, therapeutic area. Information was verified against individual NDA approval letters, labeling data, and drug summary pages to ensure accuracy and completeness. When multiple NDAs corresponded to the same active ingredient, each was treated as a distinct record if listed separately by the FDA.

## Results

A total of 45 Rx-to-OTC switches were identified between 2001 and 2025, representing 30 unique active ingredients approved by the FDA. Table 1 summarizes all FDA-approved Rx-to-OTC switch products between 2001 and 2025, detailing their therapeutic classification, route of administration, dosage form, and switch type.

The Allergy/Respiratory category accounted for the majority of all switches (25 of 45; 56%), dominated by antihistamines (loratadine, cetirizine, fexofenadine) and intranasal corticosteroids (fluticasone, mometasone, triamcinolone, budesonide). Ophthalmology and Gastrointestinal categories each contributed 11%, reflecting recent expansions of ocular antihistamines (olopatadine, alcaftadine, ketotifen) and acid reducers or laxatives (esomeprazole, lansoprazole, polyethylene glycol). Although earlier proton pump inhibitor switches, including omeprazole, represented pivotal milestones in the evolution of OTC gastrointestinal therapy, the present dataset reflects entries captured within the FDA’s current Prescription-to-Nonprescription Switch List and corresponding NDAs.

Smaller but noteworthy segments included Reproductive Health (4%), Harm Reduction/Addiction (4%), Dermatology (4%), and Antimicrobials (4%), which included topicals for acne, lice, and fungal infections. Single entries were observed for Analgesic/Anti-inflammatory (Voltaren Arthritis Pain Gel, 2%) and Urology (Oxytrol for Women, 2%).

The most frequent route was oral administration (26 of 45; 58%), encompassing antihistamines, acid reducers, and hormonal contraceptives. Nasal formulations represented 16%, largely allergy sprays. Ophthalmic and topical products each contributed 11%, while transdermal delivery accounted for 4% (nicotine and oxybutynin patches).

**Fig. 1:**
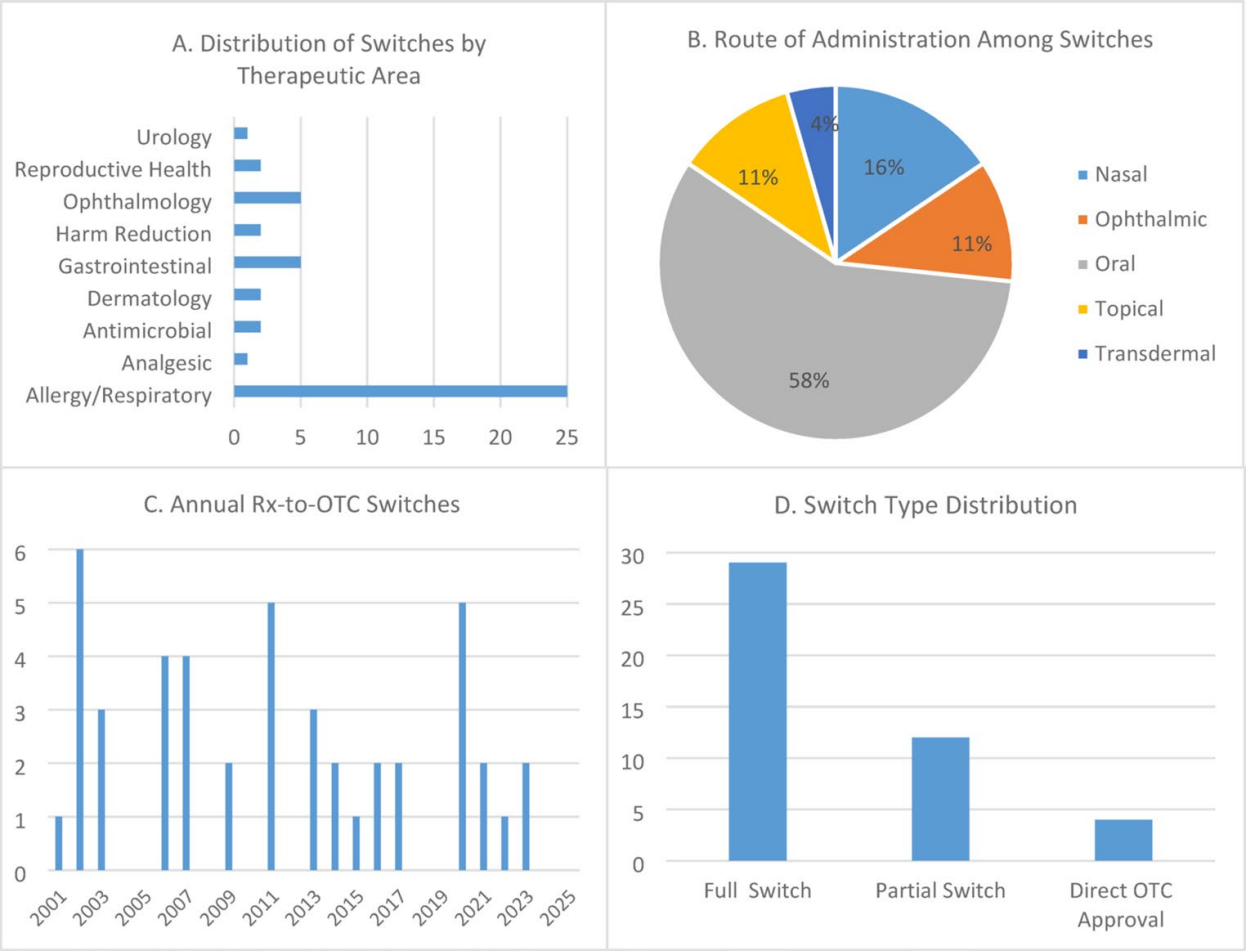
**A** Distribution of Rx-to-OTC switches by therapeutic area. **B** Route of administration among Rx-to-OTC switches. **C** Annual Rx-to-OTC switches. **D** Switch type distribution

Most products were categorized as full switches (29 of 45; 64%), reflecting complete transitions of prescription indications to OTC status. Partial switches, where only specific strengths or formulations were approved for OTC use, represented 26% (12 of 45). A small subset (9%) comprised direct OTC approval. These NDAs are not true switches since the conditions of use were not marketed as a prescription product under an approved NDA prior to being approved for marketing OTC (Fig. 1).

**Table 1:** FDA-approved Rx-to-OTC switches, 2001–2025

API	Drug name	Therapeutic area	Indication	Year	Route	Dosage form	Strength	Switch type
Norgestrel	Opill	Reproductive health	Prevention of pregnancy	2023	Oral	Tablet	0.075 mg	Full
Naloxone HCl	Narcan	Harm reduction/Addiction	Treatment of known or suspected opioid overdose, as manifested by respiratory and/or central nervous system depression	2023	Nasal	Spray	4 mg/spray	Full
mometasone Furoate	Nasonex 24 h allergy	Allergy/Respiratory	Temporarily relieves these symptoms due to hay fever or other upper respiratory allergies: nasal congestion; runny nose; sneezing; itchy nose	2022	Nasal	Spray	0.05 mg/spray	Partial
Alcaftadine	Lastacaft	Ophthalmology	Temporarily relieves itchy eyes due to pollen, ragweed, grass, animal hair and dander	2021	Ophthalmic	Solution	0.25%	Full
Azelastine HCl	Astepro allergy and children’s astepro allergy	Allergy/Respiratory	Temporarily relieves these symptoms due to hay fever or other upper respiratory allergies: nasal congestion; runny nose; sneezing; itchy nose	2021	Nasal	Spray	0.2055 mg/spray	Partial
Ivermectin	Sklice	Dermatology	Treats head lice infestation	2020	Topical	Lotion	0.50%	Full
Olopatadine HCl	Pataday once daily relief	Ophthalmology	Temporary relief of itchy eyes due to pollen, ragweed, grass, animal hair, or dander	2020	Ophthalmic	Solution	0.70%	Full
Diclofenac sodium	Voltaren arthritis pain	Analgesic/Anti-inflammatory	Temporary relief of arthritis pain: hand, wrist, elbow (upper body areas); foot, ankle, knee (lower body areas)	2020	Topical	Gel	1.00%	Full
Olopatadine HCl	Pataday twice daily relief	Ophthalmology	Temporary relief of itchy and red eyes due to pollen, ragweed, grass, animal hair, or dander	2020	Ophthalmic	Solution	0.10%	Full
Olopatadine HCl	Pataday once daily relief	Ophthalmology	Temporary relief of itchy eyes due to pollen, ragweed, grass, animal hair, or dander	2020	Ophthalmic	Solution	0.20%	Full
Levocetirizine dihydrochloride	Xyzal allergy 24 h	Allergy/Respiratory	Temporarily relieves these symptoms of hay fever or other upper respiratory allergies: runny nose; sneezing; itching of nose or throat; itchy, watery eyes (allergic rhinitis)	2017	Oral	Tablet	5 mg	Partial
Levocetirizine dihydrochloride	Xyzal allergy 24 h	Allergy/Respiratory	Temporarily relieves these symptoms of hay fever or other upper respiratory allergies: runny nose; sneezing; itching of nose or throat; itchy, watery eyes (allergic rhinitis)	2017	Oral	Solution	2.5 mg/5 ml	Partial
Fluticasone furoate	Flonase sensimist allergy relief	Allergy/Respiratory	Temporarily relieves these symptoms of hay fever or other upper respiratory allergies: nasal congestion; runny nose; sneezing; itchy nose; itchy, watery eyes (allergic rhinitis)	2016	Nasal	Spray	0.0275 mg/spray	Full
Adapalene	Differin gel	Dermatology	Anti Acne	2016	Topical	Gel	0.10%	Full
Budesonide	Rhinocort allergy spray	Allergy/Respiratory	Temporarily relieves these symptoms of hay fever or other upper respiratory allergies: nasal congestion; runny nose; sneezing; itchy nose (allergic rhinitis)	2015	Nasal	Spray	0.032 mg/spray	Full
Esomeprazole magnesium	Nexium 24 h	Gastrointestinal	Frequent heartburn	2014	Oral	Capsule	20 mg	Direct
Fluticasone proprionate	Flonase allergy relief	Allergy/Respiratory	Temporarily relieves these symptoms of hay fever or other upper respiratory allergies: nasal congestion; runny nose; sneezing; itchy nose; itchy, watery eyes (allergic rhinitis)	2014	Nasal	Spray	0.05 mg/spray	Partial
Oxybutynin	Oxytrol for women	Urology	Overactive bladder	2013	Transdermal	Film	3.9 mg/24hr	Partial
Triamcinolone acetonide	Nasacort allergy 24 h	Allergy/Respiratory	Temporarily relieves these symptoms of hay fever or other upper respiratory allergies: nasal congestion; runny nose; sneezing; itchy nose (allergic rhinitis)	2013	Nasal	Spray	0.055 mg/spray	Full
Omeprazole; Sodium bicarbonate	Zegerid OTC	Gastrointestinal	Frequent heartburn	2013	Oral	Powder	20 mg/packet;1.68 gm/packet	Direct
Fexofenadine HCl; Pseudoephedrine HCl	Allegra D 12 h	Allergy/Respiratory	Antihistamine	2011	Oral	Tablet	60 mg;120 mg	Full
Fexofenadine HCl; Pseudoephedrine HCl	Allegra 24 h	Allergy/Respiratory	Antihistamine	2011	Oral	Tablet	180 mg;240 mg	Full
Fexofenadine HCl	Allegra	Allergy/Respiratory	Antihistamine	2011	Oral	Tablet	30 mg, 60 mg, 180 mg	Full
Fexofenadine HCl	Allegra	Allergy/Respiratory	Antihistamine	2011	Oral	Suspension	30 mg/5 ml	Partial
Fexofenadine HCl	Allegra	Allergy/Respiratory	Antihistamine	2011	Oral	Tablet	30 mg	Full
Lansoprazole	Prevacid 24 h	Gastrointestinal	Acid reducer/PPI	2009	Oral	Capsule	15 mg	Direct
Omeprazole; Sodium bicarbonate	Zegerid OTC	Gastrointestinal	Acid reducer/PPI	2009	Oral	Capsule	20 mg;1.1gm	Direct
Cetirizine HCl; Pseudoephedrine HCl	Zyrtec-D	Allergy/Respiratory	Antihistamine and Nasal Decongestant	2007	Oral	Tablet	5 mg;120 mg	Full
Cetirizine HCl	Children’s Zyrtec Allergy and Children’s Zyrtec Hives Relief	Allergy/Respiratory	Antihistamine	2007	Oral	Solution	5 mg/5 ml	Partial
Cetirizine HCl	Children’s Zyrtec Allergy and Children’s Zyrtec Hives Relief	Allergy/Respiratory	Antihistamine	2007	Oral	Tablet	5 mg, 10 mg	Full
Cetirizine HCl	Zyrtec Allergy and Zyrtec Hives Relief	Allergy/Respiratory	Antihistamine	2007	Oral	Tablet	5 mg, 10 mg	Full
Terbinafine	Lamisil Derm Gel	Antimicrobial	Topical Antifungal	2006	Topical	Gel	1.00%	Partial
Levonorgestrel	Plan B	Reproductive Health	Emergency Contraceptive	2006	Oral	Tablet	0.75 mg	Full
Polyethylene glycol 3350	MiraLax	Gastrointestinal	Laxative	2006	Oral	Powder	17gm/scoopful	Partial
Ketotifen fumarate	Zaditor	Ophthalmology	Antihistamine Eye Drop	2006	Ophthalmic	Solution	0.03%	Full
Loratadine	Claritin Hives Relief Tablets	Allergy/Respiratory	Antihistamine	2003	Oral	Tablet	10 mg	Full
Loratadine	Claritin Hives Relief Reditabs	Allergy/Respiratory	Antihistamine	2003	Oral	Tablet	10 mg	Full
Loratadine	Claritin Hives Relief Solution	Allergy/Respiratory	Antihistamine	2003	Oral	Syrop	1 mg/ml	Full
Nicotine	Nicotrol TD	Harm Reduction/Addiction	Smoking Cessation	2002	Transdermal	Film	15 mg/16hr	Partial
Loratadine	Claritin Tablets	Allergy/Respiratory	Antihistamine	2002	Oral	Tablet	10 mg	Full
Loratadine	Claritin Reditabs	Allergy/Respiratory	Antihistamine	2002	Oral	Tablet	10 mg	Full
Loratadine	Claritin Solution	Allergy/Respiratory	Antihistamine	2002	Oral	Syrop	1 mg/ml	Full
Loratadine; Pseudoephedrine sulfate	Claritin-D	Allergy/Respiratory	Antihistamine/Decongestant	2002	Oral	Tablet	5 mg;120 mg	Full
Loratadine; Pseudoephedrine sulfate	Claritin-D 24-hour	Allergy/Respiratory	Antihistamine/ Decongestant	2002	Oral	Tablet	10 mg;240 mg	Full
Butenafine HCl	Lotrimin Ultra	Antimicrobial	Topical Antifungal	2001	Topical	Cream	1.00%	Partial

## Discussion

Prior analyses have described long-term trends in Rx-to-OTC switch activity in the United States, including the comprehensive evaluation by Fisher and Rawal [5]. The present study extends the publicly available FDA dataset through 2025 and characterizes switch activity by therapeutic area, route of administration, dosage form, and switch type. By incorporating recent approvals such as naloxone and oral contraceptives [8, 9], and situating these within the evolving regulatory framework, this analysis provides an updated structural perspective on the trajectory of FDA-listed Rx-to-OTC switches.

Between 2001 and 2025, 45 FDA-listed Rx-to-OTC switches representing 30 unique active ingredients were identified. The majority of approvals occurred within Allergy/Respiratory and Gastrointestinal categories, reflecting continued concentration in therapeutic areas with extensive prescription safety data and clearly defined indications suitable for self-management. Although the total number of switches appears stable relative to earlier decades, the composition of therapeutic areas demonstrates gradual expansion beyond traditional symptom-driven categories into areas with broader public health relevance.

A notable feature of the dataset is the clustering of approvals within a limited number of pharmacological classes. Multiple switches involving antihistamines and proton pump inhibitors account for a substantial proportion of entries. In several instances, distinct NDAs or supplemental NDAs correspond to the same active ingredient in different strengths, formulations, or labeling contexts. This pattern likely reflects lifecycle management strategies and incremental expansion of consumer access supported by established safety databases, rather than diversification into entirely new therapeutic mechanisms.

Full switches comprised the majority of cases (64%), indicating complete transitions of approved prescription conditions of use to nonprescription status. Partial switches accounted for 26% and were more frequently observed in therapeutically sensitive domains, including urology and hormonal products. This distribution suggests differential evidentiary and risk considerations across therapeutic areas, with partial switches serving as a regulatory mechanism to balance expanded access with risk mitigation. A small subset of approvals (9%) represented direct nonprescription NDAs listed within the FDA registry, rather than true prescription-to-nonprescription conversions.

The predominance of oral and intranasal dosage forms reflects both consumer familiarity and established patterns of self-administered therapy. Ophthalmic, topical, and transdermal products represented smaller proportions of switches. While these dosage forms have long existed within OTC monograph frameworks, recent approvals in these categories reflect incremental expansion of prescription-to-OTC availability within established delivery systems rather than structural innovation in formulation technology. Diversification therefore appears more related to therapeutic scope than to route-of-administration novelty.

It is important to recognize that FDA approval of an Rx-to-OTC switch represents the culmination of a multi-stage regulatory process. Sponsors are required to demonstrate that statutory criteria under section 503(b)(1) are satisfied, including evidence that consumers can appropriately self-select and use the product safely without practitioner supervision. Switch programs frequently involve additional clinical studies, label comprehension testing, actual-use trials, and advisory committee review. In certain high-profile cases, including emergency contraception and opioid reversal agents, broader public discourse has accompanied regulatory deliberation. Accordingly, approval trends should be interpreted as regulatory outcomes shaped by evidentiary standards and statutory requirements rather than as indicators of policy preference.

The present analysis was restricted to NDAs explicitly listed in the FDA’s Prescription-to-Nonprescription Switch List as of July 2025. Historical switches not reflected in the current registry were not independently reconstructed in order to preserve dataset consistency and reproducibility. The dataset does not capture all nonprescription innovation during the study period. Certain direct-to-OTC NDAs and products introduced through alternative regulatory pathways, including OTC Monograph Order Requests under the CARES Act modernization framework, are not systematically represented within the FDA switch list. Examples include brimonidine ophthalmic solution (Lumify), ibuprofen/acetaminophen combinations (Advil Dual Action), naloxone 3% nasal spray (RiVive), and selected sunscreen products approved under NDA pathways. Accordingly, the findings describe trends in FDA-listed Rx-to-OTC switches specifically and should not be interpreted as encompassing the entirety of OTC drug innovation.

Comparison with prior analyses should also consider differences in counting methodology. Although both the present study and Fisher et al. [5] report 45 switches, the temporal scope and inclusion criteria differ. Fisher et al. evaluated switches from 2002 to 2022 and counted multiple brand or dosage-form variants as distinct switches within their innovation framework. The present analysis extends through 2025, includes certain direct nonprescription approvals listed in the FDA registry, and reflects the composition of the switch list at the time of data extraction. These methodological distinctions, combined with the relatively modest number of new approvals between 2022 and 2025, may explain similarities in aggregate counts despite the extended timeframe.

Looking forward, the FDA’s 2024 Additional Conditions for Nonprescription Use (ACNU) framework represents an important development in the regulatory landscape [10]. ACNU permits sponsors to propose additional safeguards, including technology-assisted self-screening tools, structured questionnaires, digital labeling support, or other conditions of use, to help ensure that consumers can appropriately self-select and use certain products safely without direct healthcare supervision. This mechanism expands the traditional reliance on labeling alone and may enable consideration of therapeutic categories previously deemed unsuitable for nonprescription availability due to complexity of diagnosis, dosing, or monitoring requirements.

Emerging development programs provide early examples of how such approaches may be applied in practice. Technology-assisted self-selection models have been evaluated for statin therapy, including rosuvastatin, demonstrating the feasibility of structured digital tools to support appropriate patient selection for cardiovascular risk management [11, 12]. In parallel, ongoing actual-use studies of tadalafil 5 mg are exploring consumer ability to self-manage treatment for erectile dysfunction under nonprescription conditions [13]. These examples illustrate how ACNU-aligned strategies may support expansion of nonprescription access in therapeutic areas requiring additional safeguards beyond traditional labeling.

In early 2026, FDA Commissioner Marty Makary was quoted in a public interview stating that “everything should be over the counter that is safe,” while emphasizing that appropriate safeguards remain necessary for products requiring monitoring or risk mitigation [14]. These remarks occur alongside implementation of ACNU and ongoing modernization of OTC regulatory processes, underscoring the continued policy relevance of Rx-to-OTC switch activity [10, 15, 16]. The future trajectory of nonprescription drug policy will likely be shaped by how emerging regulatory tools, evidentiary standards, and technology-enabled safeguards are applied within existing statutory frameworks.

## Data Availability

All data analyzed in this study were derived from publicly available FDA regulatory databases, as cited in the Methods section. No original clinical datasets were generated.
